# Lupus Enteritis: Presentation as an Apparent Surgical Emergency - A Case Report

**DOI:** 10.31138/mjr.090324.ari

**Published:** 2025-05-23

**Authors:** Aarti A. Zope, Shashank M. Akerkar, Pooja G. Binnani, Prajakti S. Akerkar

**Affiliations:** 1 Department of Rheumatology, IC4 Mumbai Arthritis Clinic, Bhandup, Mumbai, Maharashtra, India;; 2 Department of Nephrology, Bethany Hospital, Thane, Maharashtra, India;; 3 K J Somaiya Hospital, Mumbai, Maharashtra, India

**Keywords:** SLE, target sign, bowel obstruction, intestinal pseudo-obstruction, lupus enteritis

## Abstract

Systemic Lupus Erythematosus (SLE) is an autoimmune disease with varied organ involvement. Gastrointestinal (GI) involvement in SLE is common, with oral mucosal lesions being the most frequently seen. Acute abdomen as a surgical emergency in SLE patients can have multiple causes. Lupus enteritis (LE)/Intestinal pseudo-obstruction (IPO) can present as acute abdomen, thereby requiring surgical reference. We hereby present a case of SLE presenting with acute abdomen due to LE/IPO, which prompted the surgeon for surgical intervention. Timely diagnosis and prompt response to steroids lead to resolution of symptoms in our case, and avoided unnecessary surgical exploration and further complications. One should keep a high degree of suspicion while managing SLE patients presenting with acute abdominal pain. Our case underscores the clinical significance of discerning lupus-related GI complications, aligning with existing literature emphasising the need for heightened clinical suspicion in acute abdominal scenarios in SLE. In this case, the patient’s acute abdomen prompted surgical consideration, reflecting the challenges in differentiating lupus-related complications from surgical emergencies.

## INTRODUCTION

Systemic Lupus Erythematosus (SLE) presents a diagnostic challenge due to its diverse manifestations. We report a compelling case where acute abdomen, initially suggestive of a surgical emergency, unveiled lupus enteritis (LE) with intestinal pseudo-obstruction (IPO) in a SLE patient. The prevalence of gastro-intestinal (GI) involvement is documented in 40 to 50% of SLE patients,^1^ with LE and IPO recognised as rare complications that demand attention due to its potential to mimic surgical conditions. Timely diagnosis of LE/IPO, supported by the documented efficacy of intravenous methylprednisolone in similar cases, averted the need for surgical intervention, aligning with the broader literature advocating for non-surgical approaches in select SLE cases. This report contributes to the existing body of knowledge by providing insights into successful non-surgical resolution of LE/IPO, thereby enhancing awareness among clinicians managing acute abdominal presentations in individuals with systemic lupus erythematosus. Our case reinforces the importance of incorporating a nuanced approach, guided by both clinical acumen and existing literature, to optimise outcomes in SLE-related GI complications. GI involvement in SLE can manifest as LE, IPO or protein-losing enteropathy. LE is characterised by inflammation of the bowel wall due to SLE activity and presents as non-specific GI symptoms. IPO is characterised by ineffective intestinal motility, intestinal obstruction without identifiable organic obstructive lesions and abdominal distension with sluggish or absent peristalsis.^2^

## CASE HISTORY

**Figure 1.**
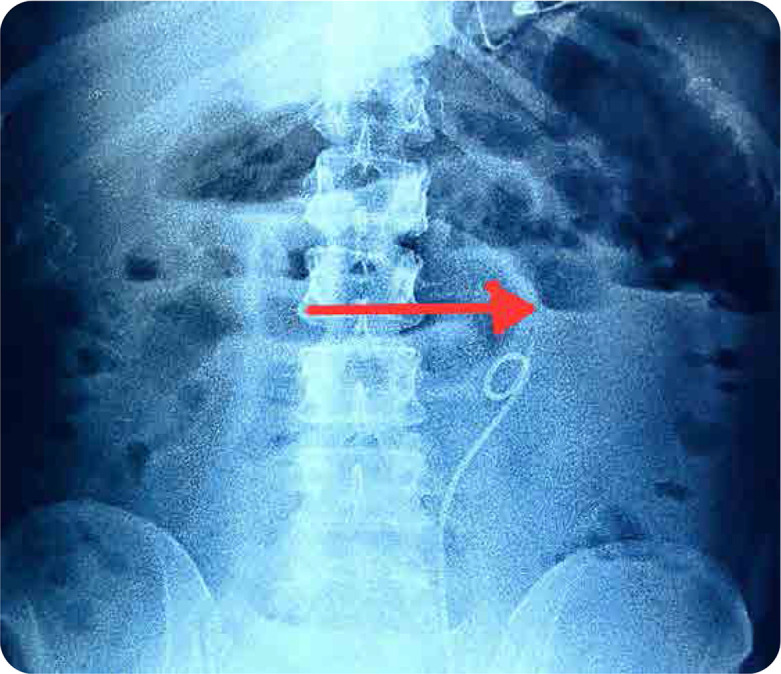
X-ray of abdomen.

**Figure 2.**
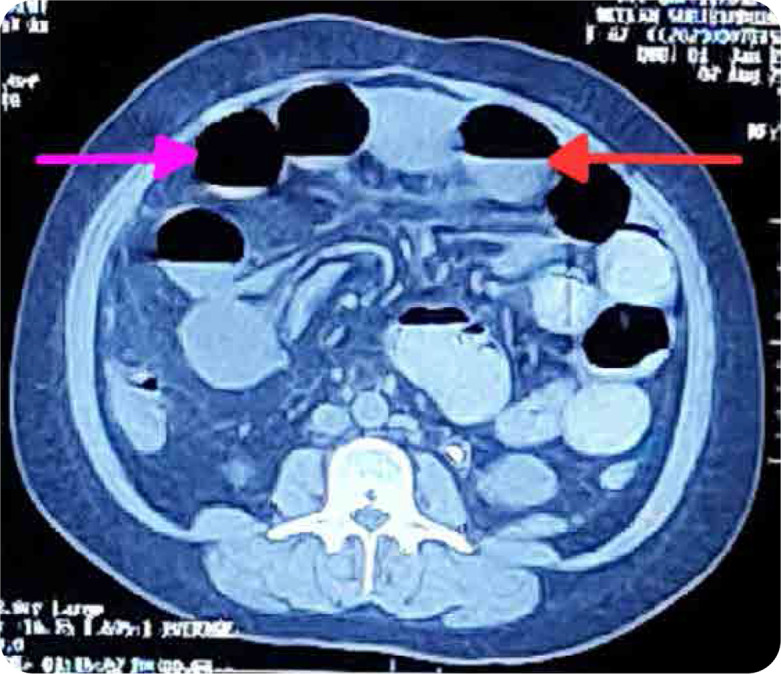
CT scan of abdomen.

A 41-year-old female, with diagnosed case of SLE, presented with acute abdominal pain, vomiting, constipation. She did not have any history of prior abdominal surgery. On examination, she had distended abdomen, with diffuse tenderness and tympanic note with sluggish bowel sounds. Her investigations revealed anaemia, leucocytosis, raised creatinine, raised C-Reactive Protein & stool showing presence of bacteria; with rest of reports being normal (**Table 1**). Her X-ray abdomen revealed prominent bowel loops in the upper abdomen and left lumbar region, with air fluid level within suggestive of bowel obstruction (**Figure 1**). She was seen by a surgeon and was started on antibiotics, Ryle’s Tube aspiration, and conservative treatment. CT scan of the abdomen revealed moderate dilatation of small bowel loops with air fluid levels within (**Figure 2**), with transition zone in infra-umbilical region; presence of small bowel faeces sign proximal to obstruction and collapse of distal ileal loops with mild ascites. Her stool and blood cultures were negative. Abdominal distension and pain persisted after seven days of conservative treatment. Her presentation and CT findings suggestive of adhesive small bowel obstruction prompted the surgeon to advise laparotomy. Historically, she was diagnosed with lupus eight years back with constitutional symptoms, musculoskeletal and muco-cutaneous involvement (fever, inflammatory polyarthritis, malar rash, positive ANA), for which she was started on oral steroids and hydroxychloroquine (HCQ). She settled, discontinued the treatment after three years and was lost to follow up. Two months before current presentation, her symptoms recurred with anasarca, oral ulcer, fever, photosensitive rash, polyarthralgia, pedal oedema, facial puffiness, and frothy urine. Her investigations revealed anaemia, thrombocytopenia, proteinuria, raised ESR, positive anti-dsDNA, and anti-Smith antibody with low C3 and C4 levels; suggestive of flare (**Table 1**). She was restarted on HCQ and symptomatic treatment and renal biopsy was planned. Renal biopsy showed Class 4 diffuse lupus nephritis. Post renal biopsy, she had abdominal pain and haematuria and was found to have bladder clot, hence underwent cystoscopy with bladder clot evacuation and ureteric stenting. One week later, she presented with above features suggestive of bowel obstruction. In view of active lupus, the possibility of LE (GI involvement secondary to SLE) with IPO was kept in mind after ruling out infectious causes. Surgical exploration was deferred and she was started on high dose IV methylprednisolone (500 mg/day for 3 days) followed by oral prednisolone (1 mg/kg/day) and mycophenolate mofetil (2 g/day). Her symptoms improved after starting intravenous pulse steroids. Repeat CT scan revealed resolution of bowel obstruction changes. During consecutive follow up over six months, her steroids were tapered to maintenance dose of 7.5 mg/day and mycophenolate mofetil was continued (2 g/day). Her abdominal symptoms had completely resolved with improvement in serum creatinine (0.8 mg/dl; normal range - 0.6 to 1 mg/dl) and proteinuria (24-hour urine protein 300 mg).

**Table 1.** Laboratory parameters at the time of admission and 2 months prior to admission.

**Parameter**	**Patient value at the time of admission**	**Patient value 2 months prior to admission**	**Reference range**
Haemoglobin (gram/dL)	8.3	8	11–14
Total leucocyte count (per mm3)	12,100	8000	4000–11000
Platelet count (per mm3)	314000	71000	150000–450000
Amylase (IU/L)	45.4	NA	25–86
Lipase (IU/L)	26.8	NA	0–60
Aspartate Transaminase (AST) (U/L)	25	20	0–40
Alanine Transaminase (ALT) (U/L)	18	14	0–50
Creatinine (mg/dL)	1.23	1.8	0.8 to 1.2
Urine routine	Normal	Protein +++, 10–12 RBCS, no casts, no bacteria	
Serum electrolytes (Sodium/Potassium) (mEq/L)	133/4.6	140/4.0	130–150/3.5–5.5
C-reactive protein (mg/L)	38.1	NA	< 6
24-hour urine protein (grams)	NA	1.9	< 0.03
Serum C3 levels	NA	48	90–180
Serum C4 levels	NA	2.9	10–40

NA: not available (reports not available).

## DISCUSSION

SLE is a chronic autoimmune disease with varied organ involvement like muco-cutaneous, musculoskeletal, haematological, renal, neurological, and others. GI symptoms are seen in 40 to 50% of SLE patients.^1^ They may be due to medication side effects, infections, peritonitis, acute pancreatitis, mesenteric thrombosis (associated with antiphospholipid antibodies), or other associated conditions like autoimmune hepatitis, primary biliary cirrhosis, inflammatory bowel disease, or celiac disease. In contrast to other autoimmune diseases, GI involvement in SLE is rare, but can be severe and life threatening. Oral mucosal lesions are most frequent, but acute abdominal pain is the most serious manifestation, which can have catastrophic presentation requiring emergent surgical intervention.^3^ Timely recognition, correct attribution to the underlying cause, and appropriate treatment are important to improve the prognosis. The diagnosis of LE is supported by either imaging or biopsy findings. It is reported in 0.2–5.8% of SLE patients.^1^ Patients with LE usually have high disease activity scores due to other organ involvement.^1^ IPO seen in 1.96% of SLE patients, with 57.6% among them presenting with it as an initial manifestation.^2^ GI involvement in SLE can be a manifestation of lupus flare (active disease) or occasionally can be a first presenting feature of the disease.

## AETIOPATHOGENESIS

LE is caused by intestinal wall inflammation secondary to the immune complex deposition and complement system activation, which can lead to vasculitis & ischemic injury. Although the postulated pathophysiology is due to small vessel arteritis and venulitis in most cases, blood vessel inflammation is not found in all patients.^4^ LE can also present as IPO, such as in our case. These patients usually present with abdominal pain, vomiting, diarrhoea, and fever, with increased risk of complications (e.g. intestinal infarction, obstruction, perforation).^4^ IPO is more common in Asians and usually presents with features suggestive of bowel obstruction without apparent mechanical obstruction. Pathogenic mechanisms postulated in IPO include systemic autoimmunity against smooth muscle cells (which explains the association with lupus cystitis and hydroureteronephrosis) and intestinal vasculitis causing visceral smooth muscle damage and visceral autonomic nervous system dysfunction.^5,6^

## CLINICAL FEATURES

Abdominal pain is the most frequent presenting feature in LE followed by ascites, nausea, vomiting, diarrhoea, and fever.^5^ Abdominal pain is seen in 8% to 40% of SLE patients.^6^ Clinical features of IPO include abdominal pain, abdominal distension, nausea, vomiting, diarrhoea, and/or constipation and weight loss. Patients may have associated features of other organ involvement of SLE, such as lupus nephritis.

## DIAGNOSIS

Abdominal X-ray in LE is usually normal, while X-ray in IPO shows dilated loops of small bowel with air-fluid levels, absence of air in pelvic cavity, stack of coins sign (suggestive of small bowel obstruction) without obvious transition point or visible obstruction.^7^ The imaging method of choice in LE is CT scan, which reveal distended bowel loops, focal or diffuse intestinal wall thickening with altered bowel wall enhancement (target sign or halo sign), engorged mesenteric vessels (comb sign), with mesenteric fat attenuation and ascites. CT scan of abdomen in IPO usually shows diffusely dilated bowel loops, bowel wall thickening, air fluid levels, and ascites, without any obvious structural obstruction, with or without associated hydroureteronephrosis.^5^ Endoscopy and colonoscopy help in identifying mucosal inflammation in LE and ruling out other diseases.^4^

### Differential Diagnosis

LE/IPO can be confused with conditions of significant morbidity and mortality, including mesenteric or hepatic thrombosis, intestinal obstruction and perforation, and other common causes of abdominal pain. Differential diagnoses of acute abdomen in SLE include Lupus mesenteric vasculitis, IPO, Acute gastroenteritis, pancreatitis, appendicitis, and cholecystitis.

## TREATMENT

LE is reversible and usually responds to high dose corticosteroids and short-term bowel rest. Refractory or relapsing disease may need second line immunosuppressive agents like azathioprine or mycophenolate mofetil. LE may be the only manifestation of active SLE requiring systemic immunosuppression, hence it is important to consider this entity as a differential diagnosis of GI symptoms in such cases. IPO is treated with high dose steroids, supportive care and immuno-suppressive agents (methotrexate, azathioprine, cyclophosphamide, IVIG). Early diagnosis can prevent unnecessary surgical intervention and repeated invasive procedures.

## COMPLICATIONS

Untreated LE patients can develop bowel ischemia, perforation, and peritonitis resulting in high mortality. Factors associated with higher recurrence rate in LE include colonic or urinary tract involvement & intestinal wall thickness > 9 mm.^4^

Lupus enteritis is being increasingly recognised and many cases have been reported in recent times. Chaparro et al. have recently reported two cases of LE. First case was a 65-year-old female, presenting with acute abdomen and diarrhoea. Further examination and workup revealed this as lupus enteritis and was the presenting manifestation of lupus. She responded well to systemic steroids and cyclophosphamide. Second case was a young female, known case of SLE, presenting with subacute abdominal complaints, with lupus nephritis flare, who was diagnosed and treated as LE, responding well to steroids and azathioprine.^8^

Iftekhar et al. recently reported a case of SLE presenting with pneumo-peritoneum without evidence of bowel perforation, responding to systemic steroids and supportive care.^9^

Naeem et al. recently reported a case of SLE presenting with LE/IPO as an initial manifestation, responding to steroids and cyclophosphamide.^10^

Thus, varied presentations of this manifestation are noted such as LE,^8,9^ IPO (similar to our case)^10^ or as a pneumo-peritoneum.^9^

This entity, once thought to be rare, is increasingly being noticed and reported. Hence, we need to keep a high degree of suspicion for presentation of lupus enteritis.

## CONCLUSION

We present a case of LE/IPO presenting as a surgical emergency. Timely diagnosis and initiation of immuno-suppressive treatment, lead to resolution of her intestinal obstruction and avoidance of major abdominal surgery. Emergency abdominal exploration would have led to unnecessary morbidity to the patient and would not have resolved the underlying pathology. Recognition of IPO due to SLE is important as it can avoid unnecessary surgical exploration, may lead to complications like perforation and is usually responsive to conservative management.

## PATIENT INFORMED CONSENT

Informed Consent was not taken from patient as the case report did not reveal any identifying features related to the patient, which may reveal her identity.

## CONFLICT OF INTEREST

The authors declare no conflict of interest.

## References

[1] Potera J Palomera Tejeda E Arora S Manadan AM. Lupus Enteritis: An Uncommon Presentation of Lupus Flare. Cureus 2021 Sep 16;13(9):e18030. doi: 10.7759/cureus.18030. PMID: 34671520; PMCID: . 34671520 PMC8520498

[2] Zhang L Xu D Yang H Tian X Wang Q Hou Y Clinical Features, Morbidity, and Risk Factors of Intestinal Pseudo-obstruction in Systemic Lupus Erythematosus: A Retrospective Case-control Study. J Rheumatol 2016 Mar;43(3):559–64. doi: 10.3899/jrheum.150074. Epub 2016 Jan 15. PMID: 26773109. 26773109

[3] Mok CC. Investigations and management of gastrointestinal and hepatic manifestations of systemic lupus erythematosus. Best Pract Res Clin Rheumatol 2005 Oct;19(5):741–66. doi: 10.1016/j.berh.2005.04.002. PMID: 16150401. 16150401

[4] Zambiasi L Zambiasi AR Tomasetto ME Bonacina P Eisenreich MA Hoppe L Lupus Enteritis: A Case Report. EMJ Gastroenterol 2023 Jun 13. doi:10.33590/emj/10308412

[5] Nyabera A Elfishawi M Cuevas F Riaz F Abrudescu A. Intestinal Pseudo-Obstruction as the Initial Clinical Presentation in Systemic Lupus Erythematosus: A Rare and Severe Disorder. Case Rep Gastrointest Med 2020 Nov 16;2020:8873917. doi: 10.1155/2020/8873917.PMID: 33274088; PMCID: . 33274088 PMC7683163

[6] Dubois. Lupus Erythematosus and Related Syndromes. 9th ed. China: Elsevier. c2019. Chapter 37, Gastrointestinal and Hepatic Systems; p. 459.

[7] García López CA Laredo-Sánchez F Malagón-Rangel J Flores-Padilla MG Nellen-Hummel H. Intestinal pseudo-obstruction in patients with systemic lupus erythematosus: a real diagnostic challenge. World J Gastroenterol 2014 Aug 28;20(32):11443–50. doi: 10.3748/wjg.v20.i32.11443. PMID: 25170234; PMCID: . 25170234 PMC4145788

[8] Chaparro CA Bernal-Macías S Muñoz OM. Lupus enteritis as systemic lupus erythematosus main manifestation: Two case reports. SAGE Open Med Case Rep 2024 Apr 15;12:2050313X241247433. doi: 10.1177/2050313X241247433.PMID: 38628859; PMCID: . 38628859 PMC11020699

[9] Iftekhar W Shaikh H Alvi AR. Systemic lupus erythematosus presenting with pneumoperitoneum without evidence of pneumatosis cystoides intestinalis-when not to operate-a case report. J Surg Case Rep 2024 Mar 27;2024(3):rjae182. doi: 10.1093/jscr/rjae182.PMID: 38549726; PMCID: . 38549726 PMC10973404

[10] Naeem F Noor MU Batool S Anwer Khan SE Akmal M. An Atypical Initial Manifestation of Systemic Lupus Erythematosus: Lupus Enteritis Accompanied by Intestinal Pseudo-Obstruction and Bilateral Hydronephroureter. Cureus 2023 Dec 16;15(12):e50628. doi: 10.7759/cureus.50628. PMID: 38226118; PMCID: . 38226118 PMC10789390

